# Assessment of the Suitability of the One-Step Hydrothermal Method for Preparation of Non-Covalently/Covalently-Bonded TiO_2_/Graphene-Based Hybrids

**DOI:** 10.3390/nano8090647

**Published:** 2018-08-23

**Authors:** Ewelina Kusiak-Nejman, Dariusz Moszyński, Joanna Kapica-Kozar, Agnieszka Wanag, Antoni W. Morawski

**Affiliations:** Institute of Inorganic Technology and Environment Engineering, Faculty of Chemical Engineering, West Pomeranian University of Technology, Szczecin, Pułaskiego 10, 70-322 Szczecin, Poland; Dariusz.Moszynski@zut.edu.pl (D.M.); Joanna.Kapica@zut.edu.pl (J.K.-K.); Agnieszka.Wanag@zut.edu.pl (A.W.); amor@zut.edu.pl (A.W.M.)

**Keywords:** TiO_2_, graphene, hybrid composites, hydrothermal method, TiO_2_-graphene bonding

## Abstract

A hybrid nanocomposites containing nanocrystalline TiO_2_ and graphene-related materials (graphene oxide or reduced graphene oxide) were successfully prepared by mechanical mixing and the hydrothermal method in the high-pressure atmosphere. The presented X-ray photoelectron spectroscopy (XPS) study and quantitative elemental analysis confirm similar content of carbon in graphene oxide GO (52 wt% and 46 wt%, respectively) and reduced graphene oxide rGO (92 wt% and 98 wt%, respectively). No chemical interactions between TiO_2_ and GO/rGO was found. TiO_2_ nanoparticles were loaded on GO or rGO flakes. However, Fourier transform infrared-diffuse reflection spectroscopy (FTIR/DRS) allowed finding peaks characteristic of GO and rGO. XPS study shows that since the concentration of TiO_2_ in the samples was no less than 95 wt%, it was assumed that the interactions between TiO_2_ and graphene should not influence the lower layers of titanium atoms in the TiO_2_ and they occurred as Ti^4+^ ions. Hydrothermal treatment at 200 °C did not cause the reduction of GO to rGO in TiO_2_-GO nanocomposites. In general, the one-step hydrothermal method must be considered to be inefficient for preparation of chemically-bonded composites synthesized from commercially available TiO_2_ and unfunctionalized graphene sheets obtained from graphite powder.

## 1. Introduction

In the last decade, the preparation and application of semiconductor nanomaterials and graphene nanocomposites have been intensively studied [1,2,3,4,5]. In the context of water and air purification, particular attention is being paid to TiO_2_-graphene hybrid nanomaterials [5,6,7,8,9,10,11]. These TiO_2_-reduced graphene oxide (TiO_2_-rGO) and TiO_2_-graphene oxide (TiO_2_-GO) nanocomposites have been synthesized with various methods [3,5] i.e., solution mixing methods, sonication-assisted mixing, sol-gel process, hydrothermal/solvothermal synthesis, self-assembly, microwave-assisted methods, direct electrochemical deposition, liquid-phase or chemical vapour deposition. 

Hydrothermal/solvothermal synthesis is commonly utilized as simple route to obtain titania-graphene hybrids. The main advantages of this process are possibility of TiO_2_ phase transformation, thermal reduction of graphene oxide to graphene (rGO), and preparation of hybrid nanocomposites with unique morphologies without post-synthetic annealing [5,12,13]. In general, preparation of TiO_2_-graphene hybrids can occur as one- or two-step hydrothermal treatment depending on whether commercially available TiO_2_ or titanium precursors is utilized. One-pot hydrothermal synthesis is commonly running with utilization of commercially available TiO_2_ and previously-prepared graphene-based materials [9,11,14,15,16,17,18]. Although the one-step synthesis of graphene-modified titania hybrids have also been conducted using titanium precursor [19,20,21], the two-step hydrothermal process is usually preceded by synthesis of titania from various titanium compounds [10,21,22,23,24,25,26].

Covalently-bonded hybrids are considered to be the most chemically stable composites. According to Bai et al. [26] the presence of strong chemical interactions through formation of Ti–O–C was possible due to preparation of TiO_2_-graphene hybrids from TiF_4_ precursor. The hydrolysis process was conducted in the presence of graphene flakes. Hydrothermal treatment was conducted to reduce GO to rGO. The appearance of Ti–O–C and Ti–C bonds was proved by means of FTIR/DRS, XPS and X-ray powder diffraction (XRD) analyses. These bonds were formed during reduction of oxygen-containing functional groups on GO edge. Liang et al. [19] synthesized TiO_2_-rGO composites conducting hydrothermal hydrolysis of Ti(SO_4_)_2_ and GO. The presence of Ti–O–C bonds was proven by the slight red-shift of the light absorption. FTIR/DRS analysis did not confirm the formation of chemical bonds between TiO_2_ and rGO. Shi et al. [21], contrary to two previous papers, confirm the non-covalent interactions in tested nanocomposites (by XPS, FTIR/DRS, and UV-VIS/DRS methods). However, in this case tertbutyl titanate and dextran were used as the TiO_2_ precursor and GO reduction medium, respectively. The reduction of GO, firstly with dextran, and then by hydrothermal treatment, was confirmed by FTIR/DRS spectroscopy.

In this work we would like to show whether preparation of chemically bonded TiO_2_-graphene materials could have been achieved with one-step hydrothermal method, utilizing commercial titania and GO or rGO sheets. Zhang et al. [14] prepared chemically-bonded TiO_2_ P25-rGO composite as a result of hydrothermal treatment of P25 and GO at 120 °C for 3 h. Ti–O–C bonds were formed during the thermal reduction of residual –COOH edge functional groups reacting with –OH groups on the surface of P25. The titania particles were mainly concentrated in the edge of graphene flakes. The chemical interactions were confirmed on the basis of FTIR/DRS analysis (suggested presence of Ti–O–C) and UV-VIS/DRS (slight bathochromic shift). Similar results were obtained by Li et al. [17]: preparation of P25-rGO at 120 °C for 3 h led to formation of chemical bonds between titania and rGO sheets during the reduction of carbon- and oxygen-containing edge functional groups of GO (confirmed by the red-shift of absorption edge). However, titania nanoparticles were located on the whole surface of graphene matrix. In contrast, Qianqian et al. [15] explained that no covalent-bonding hybrids can be prepared from commercial P25 and GO during the hydrothermal process. Titania nanotubes were loaded on the flakes of rGO (prepared from thermal reduction of GO). The slight narrowing of the band gap was related to the presence of impurities that could shift the absorption edge.

The purpose of this paper is to discuss the impact of preparation methods (mechanical mixing and hydrothermal treatment) as well as utilization of different types of graphene-related materials (GO or rGO) with various mass ratios on the structural and textural properties of hybrid nanocomposites. On the basis of obtained results the assessment of the one-pot hydrothermal method suitability for preparation of chemically-bonded hybrids form commercial TiO_2_ and graphene-related materials has been performed. In general, formation of covalent bonds between TiO_2_ and graphene-based materials is more likely to occur during hydrolysis and synthesis of TiO_2_ from precursor compounds then from commercially available titania with strictly-defined morphology.

## 2. Experimental

### 2.1. Materials and Reagents

The titanium dioxide slurry supplied by sulphate technology from Chemical Plant Grupa Azoty Zakłady Chemiczne “POLICE” S.A. (Police, Poland) was used as a bare TiO_2_ for preparation of new hybrids. Before modification the crude TiO_2_ has been pre-treated as described in our previous work [27]. Graphene materials (graphene oxide GO and reduced graphene oxide rGO) synthesized by modified Hummer’s method, were supplied by Institute of Electronic Materials Technology (Warsaw, Poland) [28]. Firstly, the appropriate amount of GO was prepared. Then, the amount of GO was divided into equal halves and one of obtained halves was reduced to obtain reduced graphene oxide. In other words, reduced graphene oxide was obtained from the same part of graphene oxide. High purity water (Millipore Elix Advantage water purification system purchased from Merck KGaA, Darmstadt, Germany, with the conductivity of 0.05 μS/cm at 25 °C) and primary aliphatic alcohol 1-butanol (purity 99%, Avantor Performance Materials Poland S.A., Gliwice, Poland) were used in order to improve the contact between titania nanoparticles and graphene sheets. BaSO_4_ (purchased from Avantor Performance Materials Poland S.A., Gliwice, Poland, purity 98%) was used as a reference material in light absorption analysis. 

### 2.2. Characterization Methods

The crystalline structure and relative phase composition of obtained materials were characterized by X-ray powder diffraction (XRD) analysis (Empyrean, Malvern Panalytical Ltd., Malvern, UK) using Cu Kα radiation (λ = 1.54056 Å). The diffractometer was equipped with a wide-angle detector (PIXcel 3D) and a monochromator, which greatly lowers the signal to noise ratio. The measurements were made in the range of 10–80° in 2θ scale. The PDF-4+ 2014 International Centre for Diffraction Data database was used for the determination of the phase composition. The average anatase crystallite diameter *d_A_* (nm) was calculated according to Scherrer’s equation. The morphology of the GO and rGO flakes, as well as the obtained selected nanocomposites, was observed by scanning electron microscopy (SEM) utilizing a Hitachi SU8020 ultra-high resolution field emission scanning electron microscope (Hitachi Ltd., Tokyo, Japan).

The Brunauer-Emmett-Teller surface area (*S_BET_*) of the obtained photocatalysts was determined on the basis of nitrogen adsorption–desorption measurements at 77 K conducted in Quadrasorb SI (Quantachrome, Boynton Beach, FL, USA) equipment. Prior to analysis, each sample was degassed at 105 °C for 12 h under high vacuum. The pore volume distributions as a function of pore size were calculated based on the Dubinin-Radushkevich equation using adsorption branches of the measured isotherm. The quantitative analysis of carbon content in GO, rGO, and TiO_2_-GO and TiO_2_-rGO samples were determined by means of a CN628 elemental analyser (LECO Corporation, Saint Joseph, MI, USA). The certified BBOT standard (LECO Corporation, Saint Joseph, MI, USA) containing 72.62 wt% of carbon, 7.35 wt% of sulphur, 6.37 wt% of nitrogen, and 6.12 wt% of hydrogen was utilized for a calibration curve for calculation of carbon concentration in GO and rGO samples. The certified EDTA standard (LECO Corporation, Saint Joseph, MI, USA) containing 41.06 wt% of carbon and 9.56 wt% of nitrogen was used for preparation of a calibration curve for calculation of carbon concentration in hybrid nanocomposites.

A 4200 Fourier transform infrared-diffuse reflection (FTIR/DRS) spectrophotometer (Jasco Co., Tokyo, Japan) equipped with diffuse reflectance accessory from the Harrick Scientific Products Inc. (Pleasantville, NY, USA) was used to characterize the TiO_2_ after modification with GO, rGO and/or a different liquid medium. 

The X-ray photoelectron experiments were performed using Al Kα (hv = 1486.6 eV) radiation with a Scienta SES 2002 spectrometer (Scienta Scientific AB, Uppsala, Sweden) operating at constant transmission energy (E_p_ = 50 eV). The spectrometer was calibrated according to the photoemission line Ag 3d_5/2_ E_B_ = 368.3 eV. The powder samples were loosely placed into the sample holder. The analysis chamber during experiments was evacuated to better than 1 × 10^−9^ mbar. Due to the charging effects observed in the obtained experimental spectra a binding energy scale had to be corrected for most of the experiments. Since all samples contained complex carbon species the adventitious carbon usually taken as a reference in XPS experiments could not be used. Since all samples contained complex carbon species the adventitious carbon usually taken as a reference in XPS experiments could not be used. The mean free path of photoelectrons in TiO_2_ is about 2 nm [29]. Therefore, a majority of the XPS signal of Ti 2p electrons originates in the second and lower layers from the surface. Since the concentration of titanium oxide in the samples was no less than 95 wt%, it was assumed that the interactions between TiO_2_ and graphene should not influence the lower layers of titanium atoms in the TiO_2_ and they occurred as Ti^4+^ ions. Regarding this, the position of the maximum of XPS Ti 2p_3/2_ line was taken as a reference position and the binding energy scale of other XPS lines acquired in the experiments were corrected accordingly. The position of XPS Ti 2p_3/2_ was arbitrarily set at 459.0 eV since previous reports indicated a range between 458.5 eV and 459.2 eV [30,31,32].

The light absorption abilities of the samples were obtained by UV-VIS-diffuse reflection spectroscopy (UV-VIS/DRS) by means of V-650 UV-VIS spectrophotometer (Jasco Co., Tokyo, Japan) equipped with an integrating sphere accessory for studying DR spectra. Barium sulphate (purity 98%, Avantor Performance Materials, Gliwice, Poland) was used as a reference material. 

All the experiments were conducted with special reference to the paper by Rogala et al. [33] discussing the influence of standard measurements on chemical and electronic structure of graphene.

### 2.3. Preparation of TiO_2_-GO and TiO_2_-rGO Nanocomposites

The tested hybrid nanocomposites were prepared in two ways: simple mechanical mixing of starting TiO_2_ (4 g) with 1 or 5 wt% of GO or rGO flakes in the agate mortar and under elevated pressure using the hydrothermal method. Firstly, 4 g of starting TiO_2_ and 40 mg (1 wt%) or 200 mg (5 wt%) of graphene oxide or reduced graphene oxide were mechanically mixed (marked with *M*) in the agate mortar. Secondly, TiO_2_ mixed with GO or rGO was placed inside BLH-800 pressure reactor (Berghof Products + Instruments GmbH, Eningen, Germany), 5 cm^3^ of ultra-pure water or butyl alcohol was added and the reactor was heated up to the programmed temperature (200 °C). The reaction suspension was kept at 200 °C for 4 h. After that time the pressure valve was opened and the sample was heated for 1 h without pressure to remove residual alcohol and water. Subsequently, the reactor was cooled down to room temperature. Then, the sample was additionally dried at 105 °C for 24 h in a vacuum drier to remove the organic carbon residues and water adsorbed on the surface of photocatalysts. The prepared materials were marked with the letter *A* in the end of the sample’s symbol. For comparison, the TiO_2_-GO and TiO_2_-rGO hybrids were thermally treated without any liquid medium (samples marked as TiO_2_-GO(1 wt%/5 wt%)-A and TiO_2_-rGO(1 wt%/5 wt%)-A).

## 3. Results and Discussion

**Figure 1:**
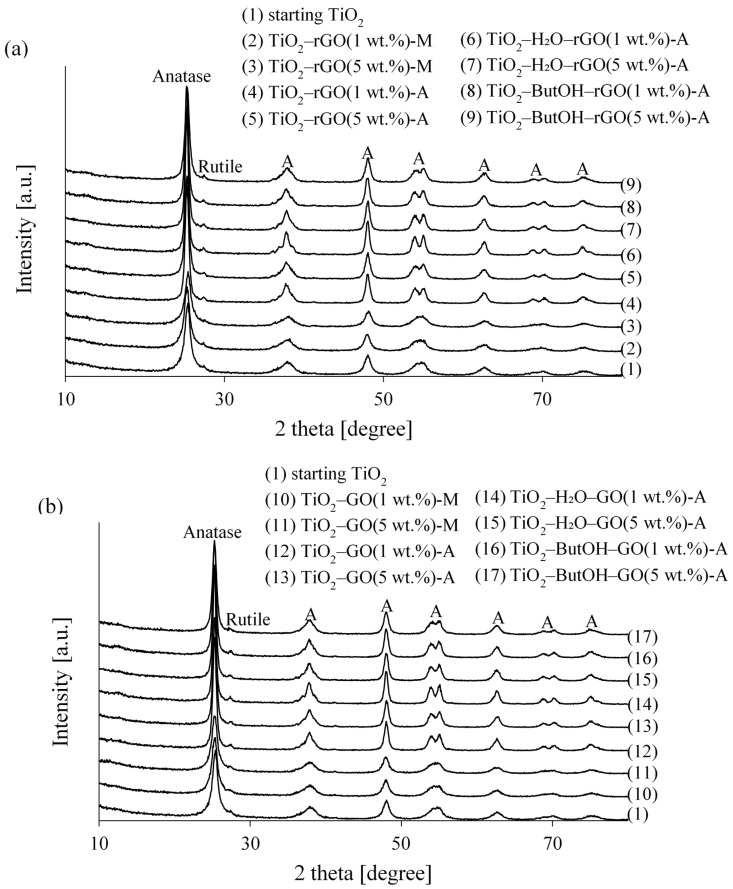
XRD patterns of (**a**) TiO_2_-rGO and (**b**) TiO_2_-GO hybrid nanocomposites.

Figure 1 presents the XRD patterns of unmodified TiO_2_, TiO_2_-GO, and TiO_2_-rGO hybrid nanomaterials. The phase composition, crystallite size, and crystallinity parameter (FWHM) of studied materials were listed in Table 1. In general, the peaks corresponding to the anatase phase and weak peak attributed to rutile (110) are observable. The rutile parameters have not been calculated due to the very low content of the rutile phase. Thus, the calculations and conclusions drawn carry a high probability of factual error. The low rutile content does not influence the properties of new composites. 

Figure 1 shows a similar diffraction peak of rGO- and GO-modified composites with starting TiO_2_. Neither thermal treatment at 200 °C nor manual mixing cause anatase-to-rutile phase transformation. Moreover, no typical diffraction peak attributed to graphene oxide and its reduced form were found due to the disappearance of the layer-stacking regularity of graphene [15,34]. The absence of a characteristic peak for rGO, independently of the amount of added rGO, located at 24.5° is due to overlap with the TiO_2_ anatase peak [35]. In addition, for TiO_2_ manually mixed with GO and rGO the increase of the FWHM parameters are observable, which may suggest the increase of the crystallinity of the anatase phase. On the other hand, the anatase crystallite size, as well as the shape of the anatase peak do not change. This suggests that the main characteristic peak of rGO is shielded by the anatase peak. It is also noted that the absence of the diffraction peak belonging to rGO was observed when commercial titanium dioxide was utilized in the preparation process [35,36]. This confirms our statement that the hydrothermal method must be considered inefficient for preparation of chemically-bonded composites from commercially available titania and unfunctionalized graphene.

Similar changes are observable for both TiO_2_ composite materials mechanically mixed with rGO and GO, namely, different amounts of carbon additives do not change the crystallinity and phase composition, which is typical for that kind of processes.

**Figure 2:**
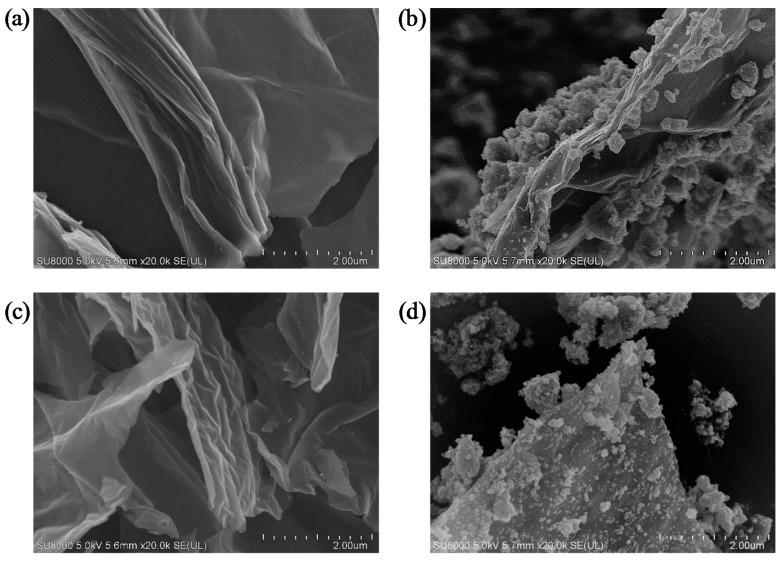
The SEM images of: (**a**) GO flake; (**b**) TiO_2_-GO(5 wt%)-A; (**c**) rGO flakes; and (**d**) TiO_2_-rGO(5 wt%)-A.

The presence of graphene in obtained nanocomposites was shown on the example of TiO_2_-ButOH-GO(5 wt%)-A and TiO_2_-ButOH-rGO(5 wt%)-A samples, as presented in the Figure 2. The SEM images also present the GO and rGO flakes to analyse the morphology of different graphene-based matrices. It can be observed that the GO flake is less than 2 μm according to the attached scale and no surface damage was found. The surface is also without any adsorbed impurities. Furthermore, it is possible to observe that the GO flake is folded and consists of a few layers (from three to four layers). The rGO morphology presented in Figure 2c shows the strongly folded structure of the mono- and bi-layer overlapped flakes. The thickness of the rGO matrix is much lower in comparison to the GO sheet. The number of layers of tested GO and rGO was also confirmed by Sobon et al. [37]. Figure 2b,d shows that the whole surface of GO or rGO sheets are decorated with TiO_2_ nanoparticles, not mainly the edges of the flakes [17].

The decrease of specific surface area (*S_BET_*) of these samples is related to the formation of TiO_2_-rGO and TiO_2_-GO aggregates. The *S_BET_* values measured for GO and rGO amount to 33 and 62 m^2^/g, respectively, which is substantially lower than the theoretical surface area (2630 m^2^/g) [38]. According to Li et al. [17] this phenomena is related with the agglomeration of graphene layers during reduction as a result of the van der Waals force between adjacent single layers of graphene. The pore volume distribution for starting TiO_2_ and modified hybrids changed insignificantly.

Thermal treatment at 200 °C generally leads to the decrease of the *S_BET_* to ca. 100 m^2^/g due to the increase of the anatase crystallite size. The shape of all peak characteristic for anatase is sharp and narrower (lower FWHM parameters) in comparison with the starting TiO_2_ and titania nanocomposites manually mixed with rGO or GO. The micropores’ volume were reduced due to thermal treatment as well as blocking by rGO and GO sheets, and the mesopores’ volume increased twice. These results show rather mesoporous nature of prepared hybrid materials.

**Figure 3:**
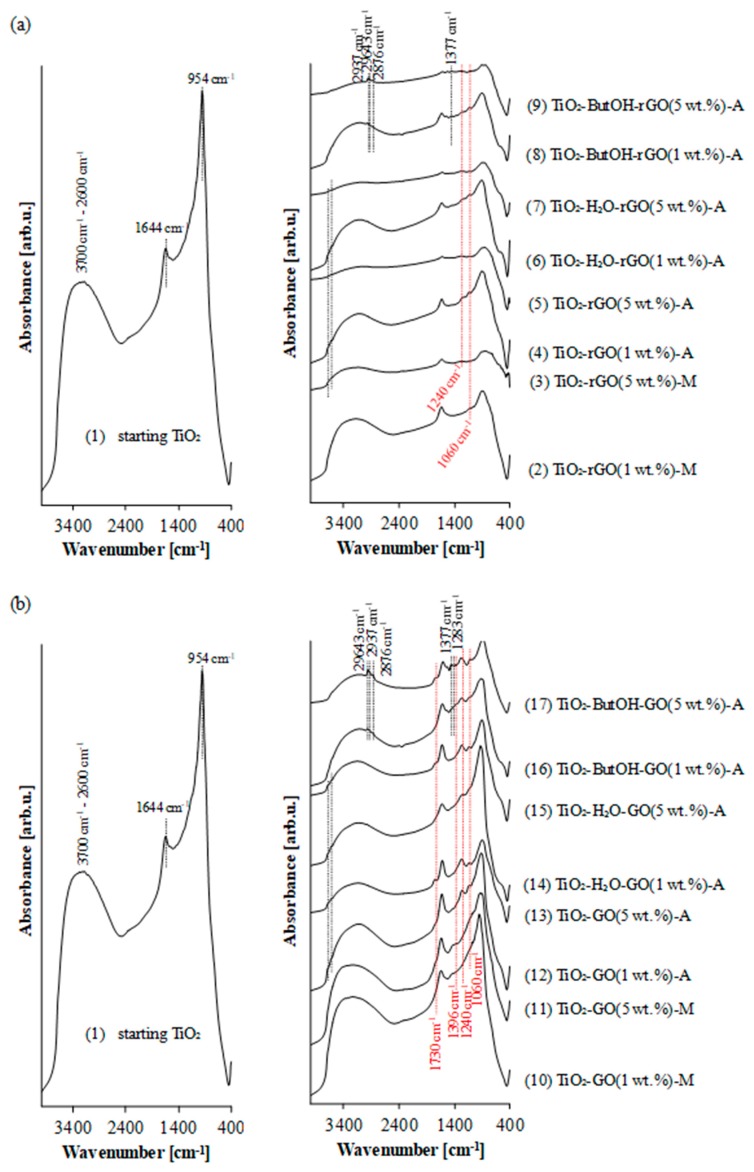
FTIR/DRS spectra of (**a**) TiO_2_-rGO and (**b**) TiO_2_-GO hybrid nanocomposite materials.

The FTIR/DRS spectra of the starting titanium dioxide and TiO_2_-GO or TiO_2_-rGO hybrid nanocomposites are presented in Figure 3. Starting TiO_2_ is characterized by the presence of a very broad peak in the range 3700–2600 cm^−1^, which is attributed to hydroxyl groups on different sites (O–H stretching of interacting hydroxyl groups, as well as the symmetric and asymmetric O–H stretching modes of molecular water coordinated for Ti^4+^ ions) [39]. For GO- and rGO-modified nanocomposites the typical broad peak located in the range 3700–3200 cm^−1^, assigned to the O–H stretching vibrations and water molecules adsorbed on the surface of carbon materials [40,41], coincides with the broad peak of TiO_2_. The characteristic narrow band at 1644 cm^−1^ is assigned to the molecular water bending mode δ_OH_ [9]. TG analysis of utilized titania (presented in our previous work) showed a small mass loss observed at 70–130 °C related to the release of moisture from the starting TiO_2_ sample [42]. The broad absorption at 954 cm^−1^ belongs to the vibration of the Ti–O–Ti and Ti–O bonds in TiO_2_ [43]. 

**Figure 4:**
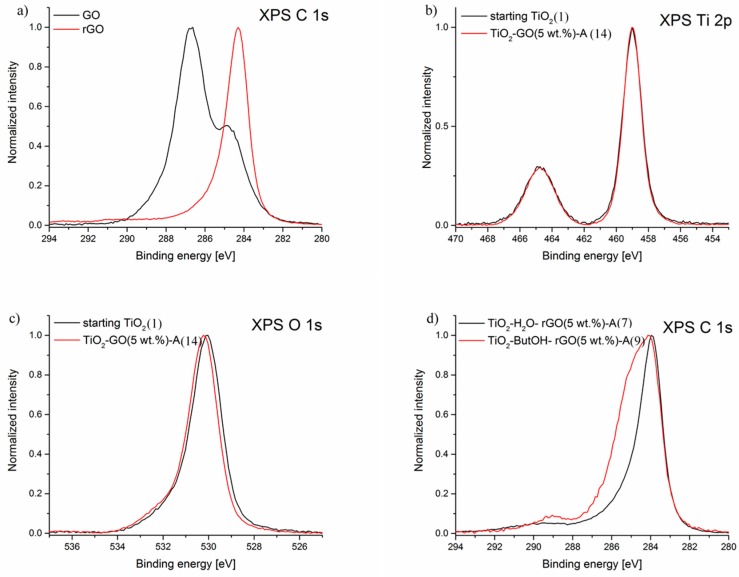

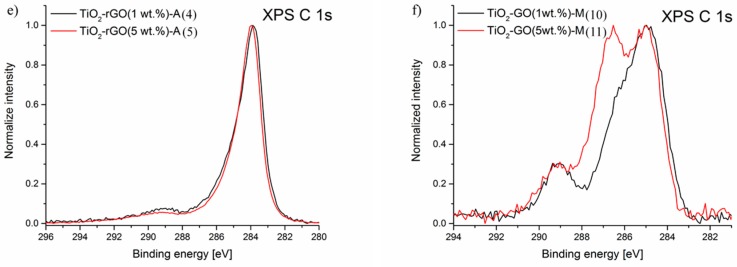
XPS spectra of: (**a**) Ti 2p line for starting TiO_2_ sample and TiO_2_-GO(5 wt%)-A nanocomposite; (**b**) O 1s line for starting TiO_2_ sample and TiO_2_-GO(5 wt%)-A nanocomposite; (**c**) C 1s line for graphene oxide (GO) and reduced graphene oxide (rGO); (**d**) C 1s line for TiO_2_-rGO composites with different solvents used; (**e**) C 1s line for samples TiO_2_-rGO treated in autoclave with different concentrations of rGO; and the (**f**) C 1s line for samples TiO_2_-GO mixed manually with different concentrations of GO.

The characteristic peaks for reduced graphene oxide at 1240 and 1060 cm^−1^ assigned to the C–O–C (epoxy) and C–O (alkoxy) stretching vibrations [40,44] respectively, are shown in Figure 3a. These peaks are observed for all modified samples, however the intensity of both is regarded as negligible. This is due to the high effective reduction of GO (studied on the basis of carbon-to-oxygen ratio) [45]. In Figure 4a the XPS spectra of reference materials: graphene oxide (GO) and reduced graphene oxide (rGO) used to form the composites with TiO_2_ are shown. Quantitative calculation of the surface composition of GO and rGO indicates that graphene oxide and reduced graphene oxide contain about 52 wt% and 92 wt% of carbon, respectively. Elemental analysis confirm the presence of 46 wt% of carbon in pure GO and 98 wt% for pure rGO in the bulk. The XPS C 1s line observed for reduced graphene oxide has a different shape from that found for GO. Its maximum is located at 284.2 eV and is characteristic for sp^2^ C–C bonds [46]. The FWHM is relatively low (1.3 eV) indicating that other components of the C 1s spectrum are not prominent. There is only small asymmetry at about 286 eV, which is attributed to minor component coming from C–O bonds [47] (possible to observe on FTIR/DRS spectra of rGO-modified materials). 

FTIR/DRS analysis of nanocomposites mixed and prepared in the autoclave shows that the intensity of peaks characteristic for TiO_2_ was lower for samples loaded with 5 wt% of rGO due to the grey colour of obtained hybrids. In addition, on the basis of FTIR/DRS analysis, some insignificant, but notable, changes in the intensity of hydroxyl groups were observed. Samples heated at 200 °C in an autoclave are characterized by a lower intensity of O–H groups at 3700–2600 cm^−1^, as well as at 1644 cm^−1^, which is typical for dehydroxylation of TiO_2_ surface due to the heat treatment [36,42,48], although the new peaks at 3688 and 3628 cm^−1^ associated with a stretching vibration modes of isolated hydroxyl groups are observed for heat-treated samples [49]. This stays in accordance with the statement that the higher amount of rGO the lower intensity of absorption peaks. 

The appearance of new peaks at 2963, 2937, and 2876 cm^−1^ assigned to the CH_3_ antisymmetric stretching, CH_2_ symmetric a stretching, CH_3_ symmetric stretching vibrations, respectively, were found for rGO and GO loaded nanocomposites obtained in the presence of 1-butyl alcohol [50,51,52,53]. The C–O peak characteristic for primary alcohols at 1050 cm^−1^ is related to C–O (alkoxy) stretching vibration at 1060 cm^−1^ for graphene sheets. 

Figure 3b presents the FTIR/DRS spectra of GO-modified TiO_2_ nanocomposites. Apart from the C–O–C (epoxy) and C–O (alkoxy) stretching bonds at 1240 and 1060 cm^−1^ characteristic for rGO, two other peaks may be observed (C=O stretching of COOH groups at 1730 cm^−1^ and C–OH at 1396 cm^−1^) [40,44]. According to XPS analysis, the main maximum of C 1s peak of GO is located at 286.7 eV and is attributed to the C–O–C bonds in the graphene oxide framework and additional local maximum at about 285 eV is attributed to the electrons from C–C sp^3^ orbitals [47,54]. A wide shoulder at the low-energy side of the spectrum presumably originates from the presence of sp^2^ C–C bonds. A similar shoulder at the high-energy side of the spectrum (at about 288 eV) can be attributed to C=O bonds. These bonds have also been found in the FTIR/DRS spectra for GO-modified TiO_2_ samples (more intensive for hybrids with 5 wt% of GO).

The FTIR/DRS analysis conducted for TiO_2_-GO composites modified in the presence of butyl alcohol shows the appearance of new bonds at 2963, 2937, and 2876 cm^−1^. These bonds are also found for TiO_2_-ButOH-rGO samples. Interestingly, the intensity of these peaks increase with the increase of GO content. It can be concluded that the molecules of alcohol has a particular affinity to the GO sheets. This corresponds to the content of carbon listed in Table 1. It was noted that for nanocomposites TiO_2_-GO(1 wt%)-A and TiO_2_-GO(5 wt%)-A the concentration of carbon amounts 0.50 and 2.50 wt%, within for TiO_2_-ButOH-GO(1 wt%)-A and TiO_2_-ButOH-GO(5 wt%)-A reaches 1.76 and 8.38 wt%, respectively. According to Wang et al. [55] the chemical bonding of TiO_2_ nanoparticles with graphene oxide is possible due to the presence of oxygen-containing functional groups mainly located on the edges of GO sheets. In contrary, Georgakilas et al. [56] postulated the presence of functional groups in GO facilitates the non-covalent functionalization of graphene sheets. The low concentration of edge functional groups may not be beneficial for the covalent bonding of GO with TiO_2_ nanoparticles [20]. 

The surface composition of the TiO_2_/graphene composites was analysed with use of X-ray photoelectron spectroscopy (XPS). On the surface of samples GO and rGO only carbon and oxygen were observed. In composites with TiO_2_ XPS signal originating from titanium atoms was also detected.

**Figure 5:**
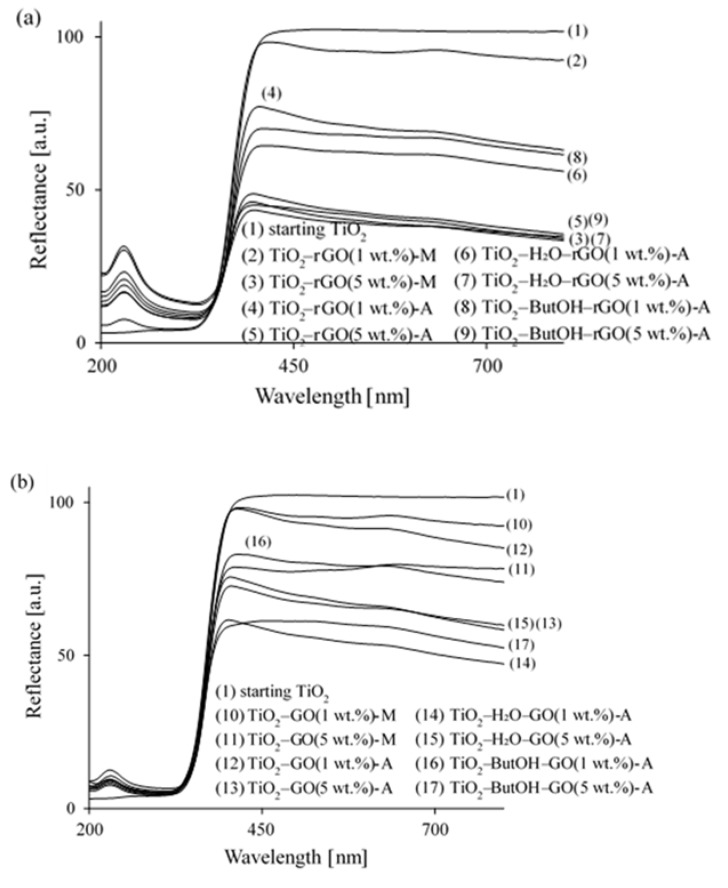
UV-VIS/DRS spectra of (**a**) TiO_2_-rGO and (**b**) TiO_2_-GO hybrid nanocomposites.

The lack of chemical interactions between TiO_2_ and utilized graphene flakes was also confirmed by means of the FTIR/DRS method. As it was discussed above, starting TiO_2_ is characterized by the presence of intensive peak at 954 cm^−1^ assigned to the vibrations of Ti–O and Ti–O–Ti framework bonds [43] (see Figure 4). After graphene modification this peak is broad with decreased intensity. This is due to the dark colour of the obtained samples, as well as the aggregation state of the graphene flakes on its surface. According to Jiang et al. [57] the presence of the Ti–O–C bond at 799 cm^−1^ proves the chemical interaction between rGO and TiO_2_. This peak was not found in our case. Additionally, the UV-VIS/DRS analysis allows to observe no red-shift of the modified TiO_2_ absorption band edge (see Figure 5). This means that no Ti–O–C and O–Ti–C were formed and the preparation process is only a physical process [15,18]. The increase of visible light absorption is strongly related with the grey and dark grey colour of obtained nanocomposites.

In Figure 4b the spectrum of the Ti 2p band for the starting TiO_2_ is compared to an example of TiO_2_/graphene composite (namely TiO_2_-GO(5 wt%)-A). Both spectra are virtually identical. Since the position of XPS Ti 2p line arbitrarily set the location of the maximum gives no information about the actual chemical state of titanium atoms. It is noteworthy that the full width at half maximum (FWHM) of that line is relatively narrow (FWHM = 1.3 eV) and the line is symmetrical. The shift between Ti 2p_3/2_ and Ti 2p_5/2_ is 5.7 eV. These observations are in line with the typical spectrum reported for pure TiO_2_. There are no visible spectral features in the ranges of about 457 eV and 455 eV, which are characteristic for Ti^3+^ ions and Ti-C bonds, which are expected if TiO_2_ interacts with carbon [58,59]. The XPS Ti 2p line for all other samples discussed in the paper are virtually identical. Consequently, the influence of graphene on the chemical state of titanium atoms is regarded as negligible. 

In Figure 4c the comparison of XPS O 1s lines for starting TiO_2_ and TiO_2_-GO(5 wt%)-A composite is given. The maximum is located at 530.0 eV and is characteristic for Ti-O bonds in TiO_2_ [31]. The very minor shift between XPS O 1s lines originating from both samples is 0.1 eV and is within an error of the correction procedure used to eliminate charging effects. The peak shape and the maximum shift magnitude observed for other samples considered in the paper are virtually identical. The maximum shift is within ± 0.1 eV for all samples. These observations support the conclusion that the TiO_2_ chemical state is not influenced by the presence of graphene.

Since no significant changes of chemical states of titanium and oxygen were observed, the surface state of the graphene is the only tool to find an insight into TiO_2_-graphene interactions. Different types of influence on the chemical state of the graphene oxide and reduced graphene oxide are expected after their mixing with TiO_2_. In Figure 4d the comparison between the chemical state of the surface of two samples of rGO mixed with TiO_2_ in autoclave are shown. They were mixed in different solvents: water (marked as TiO_2_-H₂O-rGO(5 wt%)-A) and butanol (marked as TiO_2_-ButOH-rGO(5 wt%)-A). The spectrum obtained for the sample mixed with water is virtually identical to the spectrum of pure rGO (see Figure 4a). However, the XPS C 1s spectrum coming from the sample mixed in butanol is much wider (FWHM = 2.4 eV) than the spectrum obtained for the former sample (FWHM = 1.4 eV). There is very prominent shoulder at the high-energy side of the C 1s line in the region of about 286 eV. This position is characteristic for the C 1s spectrum of alcohols. Therefore, the resulting spectrum is considered as a superposition of the signal originating from rGO covered partly with butanol. On the basis of FTIR/DRS analysis it was also noticed that the intensity of peaks at 2963, 2937, and 2876 cm^−1^ assigned to the alkyl groups increase with the increase of rGO (as well as GO) content (see Figure 3). The amount of butyl alcohol was constant for all preparation processes (5 cm^3^). This could be related to the covering of graphene flakes with butanol, as determined by the XPS measurements.

In Figure 4e the XPS spectra from TiO_2_-rGO composites with different loads of rGO are shown. In the case of reduced graphene oxide there is no observable influence of the amount of the rGO added to the mixture (1 wt% or 5 wt%). Both C 1s lines are virtually identical with the reference spectrum of rGO. There was also no difference in the surface composition of the TiO_2_-rGO composites considering different mixing type, manual or in the autoclave. However, the FTIR/DRS analysis confirm the increase of the intensity of peaks characteristic for C–O and C–O–C bonds for samples loaded with 5 wt% of rGO.

Considering mixing of TiO_2_ with graphene oxide much more prominent changes are observed. In Figure 4e the chemical state of carbon is compared for two samples mixed manually with different amounts of GO. The spectrum of the TiO_2_-GO(1wt%)-M sample has a maximum at about 285 eV and its envelope is very similar to the adventitious carbon observed on the pure TiO_2_. The envelope of the XPS C 1s line coming from TiO_2_-GO(5 wt%)-M sample is much different. There are two main maxima. The first is located at about 285 eV and the second at 286.7 eV. The second maximum is identical with the maximum observed for the reference spectrum of GO (see Figure 4a). Therefore, the former spectrum is considered as a superposition of XPS C 1s signal coming from the surface of TiO_2_ covered with adventitious carbon and XPS C 1s signal originating from GO. This can indicate that the manual mixing of GO with TiO_2_ is inefficient. Mixing of GO with TiO_2_ in the autoclave results in the almost identical spectrum for both small and high concentration of GO and are both similar to the spectrum of TiO_2_-GO(1 wt%)-M sample. 

It is also worth mentioning that thermal treatment in a high-pressure atmosphere does not cause the reduction of GO to rGO. It has been reported in previously-published papers that the decomposition of oxygen-containing functional groups in GO occurs at ca. 200 °C [19,57,60]. In this case, the FTIR/DRS as well as XPS analysis for TiO_2_-GO(1 wt%)-M and TiO_2_-GO(1 wt%)-A confirm the presence of several groups characteristic for graphene oxide. On the basis of quantitative calculation of the surface composition and quantitative elemental analysis of the above-mentioned samples, no significant changes in carbon content before and after thermal treatment was found. It could be concluded that the hydrothermal reduction of GO did not occur. However, XPS study for TiO_2_-GO(5 wt%)-A confirms almost total reduction of GO in the tested sample (C 1s signal very similar to TiO_2_-rGO(5 wt%)-A). Hence, the amount of 1wt% of carbon is to less to observe changes typical for thermal reduction of GO to rGO. Interestingly, the FTIR/DRS analysis for TiO_2_-GO(5 wt%)-A proves the presence of typical peaks characteristic for GO. These results suggest the surface reduction of GO, thus, no bulk-reduction occurs.

The light absorption abilities of tested hybrids were obtained by UV-VIS-diffuse reflection spectroscopy (UV-VIS/DRS absorption spectra presented in Figure 5). The starting TiO_2_ displays a typical absorption with an intense transition in UV region (assigned to the intrinsic band gap absorption of titania) [22]. For both kinds of modification (with GO and rGO) slight change of band gap energy was noticed. This means that no carbon doping occurred, as mentioned previously. Qianqian et al. [15] concluded that an insignificant bathochromic shift occurs because of the impurity level. The GO or rGO sheets were decorated with TiO_2_ nanoparticles [36]. In addition, the absorption peak at 230 nm attributed to the π-π* plasmon peak (corresponding to the aromatic C=C single bond) was found for GO- [61,62] and rGO-modified materials. For GO-loaded composites no absorption peak at 290–310 nm corresponding to a n-π* plasmon peak was possible to observe due to the high UV absorption of TiO_2_. According to Saxena et al. [63] this n-π* plasmon peak is characteristic for epoxide C–O–C and peroxide R–O–O–R like linkages. Kumar et al. [64] have assigned the n-π* transition to the C=O bonds, which could disappear or decrease in intensity upon reduction due to the elimination of C=O bonds. The C–O–C, as well as C=O bonds, were analysed by means of both FTIR/DRS and XPS spectroscopy. Interestingly, no red-shift of 230 nm bond is observed for rGO-loaded hybrids. This red-shift to 260–290 nm is characteristic for reduction of graphene oxide due to an increase in the restored carbon network conjugation length [62,65,66]. However, some reduction procedures, e.g., thermal treatment in water, do not result in a red-shift of π-π* plasmon peak [67].

Enhanced visible light absorption occurs due to the colour changes of hybrids modified with GO or rGO changed the colour from white to grey and dark grey, respectively [65,68]. The starting TiO_2_ strongly reflects visible light due to the white colour. Gurunathan et al. [69] concluded that the black colour of the reduced graphene oxide indicates the deoxygenation resulting in electronic conjugation within the reduced sheets. In this case, UV-VIS/DRS analysis does not offer a clear answer to the question of whether the GO was reduced successfully (confirmed by means of elemental analysis, FTIR/DRS and XPS).

## 4. Conclusions

Summarizing, this paper presents the characterization of structural and textural properties of TiO_2_ hybrid nanocomposites modified with different graphene-related materials (reduced graphene oxide and graphene oxide) prepared by the hydrothermal method in a high-pressure atmosphere. Synopsizing information presented in this article, the following general conclusions can be drawn:

- Preparation method: the hydrothermal method must be considered to be inefficient for preparation of chemically-bonded composites synthesized from commercially available TiO_2_ and unfunctionalized graphene-related sheets (prepared from graphite). Hydrothermal treatment at 200 °C caused a reduction of GO to rGO in TiO_2_-GO nanocomposites, however, this phenomena was observed for samples containing 5 wt% of GO.
- Interactions: lack of chemical bands between TiO_2_ and utilized graphene materials is due to the application of commercial titania from sulphate technology. TiO_2_ nanoparticles were loaded on GO or rGO flakes;
- Chemical bonding analysis: utilization of at least XPS, FTIR/DRS, and UV-VIS/DRS methods is sufficient to prove the presence of chemical interactions between TiO_2_ and graphene-based materials. All analysis must be conducted with enhanced caution due to the strong influence of standard measurements on the chemical and electronic structures of graphene.

## Figures and Tables

**Table 1:** The physicochemical properties of TiO_2_-GO and TiO_2_-rGO nanocomposites.

Sample Code	Anatase Content (%)	Anatase Parameters		*S_BET_* (m^2^/g)	*V_total 0.99_* (cm^3^/g)	*V_micro DR_* (cm^3^/g)	*V_meso_* (cm^3^/g)	Carbon Content (wt%)
		d_A_* (nm)	FWHM (°)					
starting TiO_2_	99	12	0.79	312	0.27	0.11	0.16	0
TiO_2_-rGO(1 wt%)-M	99	11	0.87	281	0.29	0.10	0.19	1.05
TiO_2_-rGO(5 wt%)-M	99	10	0.93	268	0.27	0.10	0.17	4.54
TiO_2_-rGO(1 wt%)-A	99	18	0.56	94	0.26	0.03	0.23	0.89
TiO_2_-rGO(5 wt%)-A	99	16	0.61	106	0.26	0.04	0.22	4.92
TiO_2_-H₂O-rGO(1 wt%)-A	99	22	0.49	83	0.35	0.03	0.32	1.07
TiO_2_–H₂O-rGO(5 wt%)-A	99	20	0.52	87	0.29	0.03	0.26	4.80
TiO_2_–ButOH-rGO(1 wt%)-A	99	19	0.53	103	0.33	0.04	0.29	1.62
TiO_2_–ButOH-rGO(5 wt%)-A	99	15	0.66	111	0.27	0.04	0.23	6.27
TiO_2_-GO(1 wt%)-M	99	11	0.88	253	0.29	0.09	0.20	0.50
TiO_2_-GO(5 wt%)-M	99	11	0.88	266	0.37	0.12	0.25	1.80
TiO_2_-GO(1 wt%)-A	99	18	0.55	93	0.32	0.03	0.29	0.44
TiO_2_–GO(5 wt%)-A	99	18	0.56	98	0.31	0.04	0.31	2.50
TiO_2_–H₂O–GO(1 wt%)-A	99	22	0.47	86	0.35	0.03	0.32	0.56
TiO_2_–H₂O–GO(5 wt%)-A	99	19	0.54	100	0.33	0.04	0.29	1.99
TiO_2_–ButOH–GO(1 wt%)-A	99	18	0.56	99	0.38	0.04	0.34	1.76
TiO_2_–ButOH–GO(5 wt%)-A	99	15	0.67	122	0.32	0.04	0.28	8.38

*—measured at 2θ = 25.4°; M—mechanical mixing; A—autoclave.
